# Baicalin Alleviates ADAM17/EGFR Axis-Induced Peritonitis in Weaned Piglets Infected by *Glaesserella parasuis*

**DOI:** 10.3390/ani15162457

**Published:** 2025-08-21

**Authors:** Qirong Lu, Xuwen Liu, Junke Tian, Pu Guo, Chun Ye, Shulin Fu, Yu Liu, Yinsheng Qiu

**Affiliations:** 1 Hubei Key Laboratory of Animal Nutrition and Feed Science, School of Animal Science and Nutritional Engineering, Wuhan Polytechnic University, Wuhan 430023, China; qirongluvet@whpu.edu.cn (Q.L.);; 2 Wuhan Engineering and Technology Research Center of Animal Disease-Resistant Nutrition, School of Animal Science and Nutritional Engineering, Wuhan Polytechnic University, Wuhan 430023, China

**Keywords:** Glässer’s disease, traditional Chinese medicine, antibiotic resistance, anti-inflammatory effects, small interfering RNA, swine

## Abstract

*Glaesserella parasuis* (*GPS*) is a Gram-negative, pathogenic bacterium that colonizes the upper respiratory tract of piglets and causes Glässer’s disease with peritonitis under stress conditions. The mechanism underlying GPS-induced peritonitis in piglets remains unclear. Baicalin is one of the main active ingredients of Huangqin (*Scutellaria baicalensis*), which has a significant anti-inflammatory effect on inflammatory diseases. Therefore, this study aimed to elucidate the molecular mechanism by which baicalin alleviates *GPS*-induced peritonitis in piglets, specifically focusing on the role of the ADAM17/EGFR signaling axis. We investigated the effects of baicalin in vitro using porcine peritoneal mesothelial cells (PPMCs) and in vivo in GPS-infected piglets. Our results showed that baicalin reduced the expression of the pro-inflammatory cytokines tumor necrosis factor alpha (TNF-α), interleukin-1 beta (IL-1β), and interleukin-6 (IL-6) in PPMCs and the peritoneum of piglets after *GPS* infection. Concurrently, baicalin significantly reduced the upregulation of disintegrin and metalloproteinase 17 (ADAM17), phosphorylated epidermal growth factor receptor (p-EGFR)/EGFR, and phosphorylated extracellular signal-regulated kinase (p-ERK)/ERK induced by *GPS* infection in PPMCs and the peritoneum of piglets. Crucially, in vitro mechanistic investigations revealed that baicalin can significantly reduce the upregulation of ADAM17, p-EGFR/EGFR, p-ERK/ERK, TNF-α, IL-1β, and IL-6 induced by ADAM17 overexpression in PPMCs. Furthermore, ADAM17 small interfering RNA can significantly reduce the upregulation of ADAM17, p-EGFR/EGFR, p-ERK/ERK, TNF-α, IL-1β, and IL-6 induced by *GPS* infection in PPMCs. These findings demonstrate that baicalin can inhibit the expression of inflammatory factors TNF-α, IL-1β, and IL-6 through the ADAM17/EGFR axis, and then alleviate the peritonitis caused by *GPS* in piglets. This provides a theoretical basis for developing novel non-antibiotic strategies, including phytochemical therapeutics and feed additives, for preventing and controlling GPS.

## Simple Summary

*Glaesserella parasuis* (*GPS*), a Gram-negative bacterium that colonizes piglets’ upper respiratory tracts, causes Glässer’s disease including peritonitis under stress, though the peritonitis mechanism remains unclear. Baicalin, a main active component of Huangqin (*Scutellaria baicalensis*), exhibits significant anti-inflammatory effects. This study aimed to investigate the mechanism by which baicalin alleviates *GPS*-induced peritonitis in piglets. We examined the effects of baicalin on inflammatory responses and key signaling molecules in both porcine peritoneal mesothelial cells (PPMCs) in vitro and the peritoneum of *GPS*-infected piglets in vivo. Our results showed that baicalin significantly reduced *GPS*-induced expression of pro-inflammatory cytokines (tumor necrosis factor alpha, interleukin-1 beta, and interleukin-6) and inhibited the upregulation of disintegrin and metalloproteinase 17 (ADAM17), phosphorylated epidermal growth factor receptor (EGFR)/EGFR, and phosphorylated extracellular signal-regulated kinase (ERK)/ERK. Mechanistically, baicalin inhibited ADAM17 overexpression-induced upregulation of this pathway and cytokines in PPMCs, while ADAM17 small interfering RNA attenuated *GPS*-induced effects. These findings demonstrate that baicalin mitigates *GPS*-induced peritonitis by suppressing the ADAM17/EGFR axis and the subsequent cytokine production, thus providing a theoretical basis for novel *GPS* control strategies and the development of novel non-antibiotic strategies, including phytochemical therapeutics and feed additives.

## 1. Introduction

*Glaesserella parasuis* (*GPS*) is a Gram-negative, pathogenic bacterium that colonizes the upper respiratory tract of pigs [1,2]. Pigs infected with *GPS* may exhibit Glässer’s disease, which is characterized by fibrinous polyserositis, meningitis, and polyarthritis [3,4], leading to large economic losses in the swine industry worldwide [5]. At present, antibiotics and vaccines are often used to control *GPS* infection, but there are reports of antibiotic resistance in clinical *GPS* isolates, and the main problem of the commercial *GPS* vaccines is the low degree of cross-protection against multiple heterologous serotypes [6,7]. This low cross-protection between commercial vaccines and *GPS* isolates has led to the practice of using stock-specific vaccines derived from isolated strains within many pig herds [6,8,9,10]. However, not all isolated *GPS* isolates are suitable for such a vaccine. Given that the disease is widely prevalent, causing huge economic losses, it is urgent to further study the pathogenic mechanism of *GPS* infection in piglets and the measures to prevent and control *GPS* infection.

Polyserositis is a multi-site inflammatory and exudative disease that affects the pericardium, pleura, and peritoneum [11]. Peritonitis is a specific manifestation of serositis in the abdominal cavity and a common clinical symptom after *GPS* infection in piglets [12,13,14]. Previous research has reported that *GPS* infection can induce apoptosis via the protein kinase C-mitogen-activated protein kinases (PKC-MAPK) pathway in porcine peritoneal mesothelial cells (PPMCs) [3]. *GPS* infection may disrupt the peritoneal tight junctions in piglets by downregulating the expression of the tight junctions’ proteins [12]. Currently, the mechanism of peritonitis induction by *GPS* infection in piglets is still unclear. Numerous studies have shown that *GPS* infection can induce inflammatory responses in piglets [15,16,17]. Therefore, exploring the mechanism of peritonitis induction from the perspective of inflammation may be an effective measure to prevent and control peritonitis caused by *GPS* infection.

Research efforts are increasingly focused on non-antibiotic alternatives, such as plant chemical extracts, feed additives, prebiotics, and enzyme preparations, for enhancing health and preventing disease in livestock and poultry [18,19,20,21]. Baicalin is an effective ingredient of the Chinese traditional medicine Huangqin (*Scutellaria baicalensis*), which is widely used in the clinical treatment of inflammatory injuries [22,23,24]. Baicalin regulates the toll-like receptor 6-mediated nuclear factor kappa B (NF-κB) signaling pathway to alleviate *Mycoplasma gallisepticum*-induced lung inflammation in chickens [25]. Baicalin alleviates NOD-like receptor pyrin 3 inflammasome (NLRP3) and NF-κB signaling in piglet mononuclear phagocytes during *GPS* infection [26] and inhibits the release of high mobility group box 1 (HMGB1) protein in peripheral blood monocytes induced by *GPS* infection [27]. The potential for baicalin to alleviate peritonitis caused by *GPS* infection in piglets has not been studied.

In this study, we evaluated the regulatory effect of baicalin on peritonitis caused by *GPS* infection in vivo and in vitro. Our results showed that baicalin has an alleviating effect on inflammatory injury of the peritoneum in piglets infected with *GPS*, providing a theoretical basis for clinical relief of peritonitis caused by *GPS* infection.

## 2. Materials and Methods

### 2.1. Reagents and Chemicals

Baicalin (CAS No. 21967-41-9; purity ≥ 98%) was purchased from Aladdin (Shanghai, China). Dimethyl sulfoxide (DMSO) (CAS No. 67-68-5; purity ≥ 99%) was purchased from Yeasen (Shanghai, China).

### 2.2. Cell and Bacterial Cultures

PPMCs between passages 3 and 6 were maintained in DMEM/F-12 medium (Cytiva, Marlborough, MA, USA) supplemented with 2% fetal bovine serum (PAN Biotech, Aidenach, Germany) and 1% penicillin–streptomycin (Gibco, New York, NY, USA). The cells were incubated at 37 °C under a humidified atmosphere containing 5% CO_2_.

Prior to infection, the GPS strain SH0165 (serovar 5) was routinely cultured in tryptic soy broth (TSB) (Hopebio, Qingdao, Shandong, China) or on tryptic soy agar (Hopebio, Qingdao, Shandong, China) at 37 °C. Both media were supplemented with 10% newborn calf serum (ZhejiangTianhang Biotechnology Co., Ltd., Huzhou, Zhejiang, China) and 1% nicotinamide adenine dinucleotide (Sigma, Saint Louis, MO, USA). The strain was passaged every 12 h in TSB; purity was confirmed by Gram staining and microscopy, showing uniform bacterial morphology; and viability was determined by counting colony-forming units (CFUs) on agar plates, with a viability rate consistently exceeding 95%. The *GPS* strain was quantified by measuring its optical density at 600 nm (OD_600_) using a spectrophotometer (MAPADA, Shanghai, China).

### 2.3. Experiment Design

A total of 40 21-day-old naturally farrowed early-weaned piglets (Duroc × Landrace × Large White, with equal numbers of males and females (20 each)) with initial body weights of 5–6 kg were obtained from the Wuhan Fenglongxin Breeding Professional Cooperative (Wuhan, Hubei, China). The piglets tested negative for antibodies against *GPS*. Upon arrival, the piglets were acclimated for 7 days under controlled conditions: the temperature was maintained at 28–30 °C, and ad libitum access to water and a standard starter diet were provided. They were housed on raised plastic flooring with wood shavings available as bedding. Following acclimation, the piglets were randomly allocated by block randomization, to ensure balanced distribution of sex across groups, to five experimental groups (*n* = 8/group) as follows: control group without *GPS* infection; infection without baicalin treatment group; baicalin at 25 mg/kg of body weight (b.w.) group; baicalin at 50 mg/kg b.w. group; and baicalin at 100 mg/kg b.w. baicalin group. The baicalin-treated groups received intramuscular injections of the appropriate concentrations 2 h prior to *GPS* infection. All groups except the control group were intraperitoneally administered 1 mL of TSB containing 1 × 10^8^ CFUs of *GPS*. The control group received an equivalent volume of sterile TSB. Six hours post-inoculation, the baicalin-treated piglets were injected with the appropriate dosage again. Subsequently, each baicalin treatment was administered twice a day for two consecutive days. Following *GPS* inoculation, the piglets were monitored for 7 d. Body temperature, body weight, and survival rate were recorded. The piglets were then euthanized via intravenous injection of sodium pentobarbital followed by exsanguination. The peritoneal tissue of piglets was obtained from our previous animal experiment [28]. Peritoneal tissue samples were collected, rapidly frozen in liquid nitrogen, and stored at −80 °C for molecular analysis. All experimental procedures were approved by the Animal Care and Use Committee of Wuhan Polytechnic University, Hubei Province, China (Approval No. WPU202308004).

### 2.4. Cell Viability Assay

PPMCs were plated in 96-well culture plates at a density of 2 × 10^4^ cells/well and then exposed to varying concentrations of baicalin (6.25–800 μg/mL) for 12 h. After treatment, PPMC viability was assessed using a cell counting kit-8 (CCK8) kit (Vazyme, Nanjing, Jiangsu, China) according to the manufacturer’s standardized protocol. Absorbance measurements were performed at 450 nm with a microplate reader (Spectra MIX i3x, Molecular Devices, Shanghai, China).

### 2.5. Cell Transfection

For the disintegrin and metalloproteinase 17 (ADAM17) overexpression, PPMCs were transiently transfected with the recombinant plasmid pcDNA3.1(+)-ADAM17 (1 µg per well) or the empty vector control pcDNA3.1(+) (1 µg per well) using lipo8000™ transfection reagent (Beyotime, Shanghai, China) according to the manufacturer’s standardized protocol. The transfection complex was removed and replaced with fresh complete medium after 4 h of exposure. Cells were then treated with various concentrations of baicalin for an additional 12 h. Protein was subsequently harvested and ADAM17 overexpression efficiency was assessed by Western blot analysis. For RNA interference (RNAi) targeting ADAM17, PPMCs were transiently transfected with small interfering RNA (siRNA) targeting ADAM17 (20 nM final concentration; GenePharma, Suzhou, Jiangsu, China) or negative control siRNA (NC) (20 nM final concentration; GenePharma, Suzhou, Jiangsu, China) using the lipo8000™ transfection reagent (Beyotime, Shanghai, China) following the manufacturer’s standardized protocol. Cells were exposed to the transfection complexes for 4 h, followed by replacement with fresh complete medium for a further 12 h. Subsequently, cells were treated with *GPS* at a multiplicity of infection (MOI) of 100 for 12 h. Protein was subsequently harvested and ADAM17 knockdown efficiency was evaluated by Western blot analysis.

### 2.6. Western Blot

Western blot analysis was performed as follows. Protein samples extracted from cells and tissues were loaded at 20 µg per lane, separated by 10% sodium dodecyl sulfate polyacrylamide gel electrophoresis (Sangon Biotech, Shanghai, China), and subsequently transferred onto polyvinylidene fluoride membranes by using a semi-dry transfer system (WIX-fastBLOT, Beijing, China). The membranes were blocked with 5% nonfat milk or 5% bovine serum albumin (BSA) in Tris-buffered saline containing 0.1% Tween-20 (TBST), followed by incubation with the following specific primary antibodies at 4 °C overnight: ADAM17 antibody (Bioss, Beijing, China; 1:1000); epidermal growth factor receptor (EGFR) antibody (PTM BIO, Hangzhou, Zhejiang, China; 1:1000); extracellular signal-regulated kinase (ERK) antibody, phosphorylated ERK (p-ERK) antibody, and tumor necrosis factor alpha (TNF-α) antibody (Proteintech, Wuhan, Hubei, China; 1:1000); phosphorylated EGFR (p-EGFR) antibody, interleukin 1 beta (IL-1β) antibody, and interleukin 6 (IL-6) antibody (Abclonal, Wuhan, Hubei, China; 1:1000); glyceraldehyde-3-phosphate dehydrogenase (GAPDH) antibody (Servicebio, Wuhan, Hubei, China; 1:5000). After three washes with TBST, the membranes were incubated with horseradish peroxidase-conjugated secondary antibody (Abclonal, Wuhan, Hubei, China; 1:8000). Finally, the protein bands were visualized with an enhanced chemiluminescence kit (Abclonal, Wuhan, Hubei, China) and imaged with a FluorChem E imaging system (ProteinSimple, San Jose, CA, USA) using a standardized exposure time of 30 s-5 min, optimized to avoid saturation. Band intensity quantification was performed using Image J software (National Institutes of Health, Bethesda, MD, USA), with normalization conducted as follows: p-ERK band intensities were normalized to total ERK, p-EGFR to total EGFR, and all other protein bands to GAPDH.

### 2.7. Statistical Analysis

Summarised data are presented as the mean ± standard deviation (SD). Statistical analyses were performed using a one-way analysis of variance followed by Duncan’s post hoc analysis. Significant and strongly significant differences were considered at *p* < 0.05 and *p* < 0.01, respectively.

## 3. Results

### 3.1. Effect of Baicalin Concentration on Cytotoxicity to PPMCs

**Figure 1:**
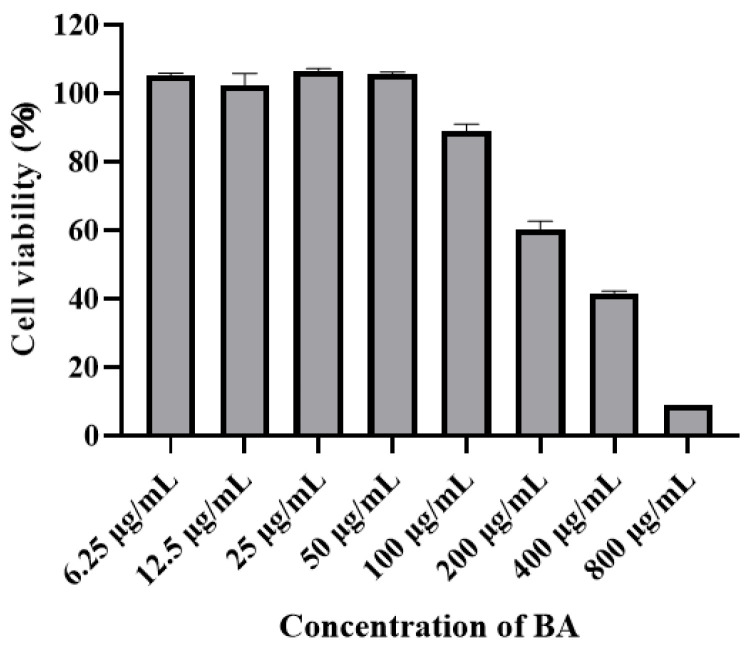
The cytotoxicity of baicalin to porcine peritoneal mesothelial cells. The cells were treated with 6.25–800 μg/mL of baicalin for 12 h. BA stands for baicalin.

To explore baicalin’s cytotoxicity to PPMCs at different concentrations, a CCK8 assay was used. The cytotoxicity of baicalin to PPMCs showed a concentration-dependent trend. The higher the concentration of baicalin, the lower the cell survival rate and the greater the cytotoxicity. When the concentration of baicalin exceeded 100 μg/mL, the cell survival rate decreased to less than 80%, which was considered significant cytotoxicity (Figure 1).

### 3.2. Baicalin Inhibition of Inflammatory Protein Expression in PPMCs Infected with GPS

**Figure 2:**
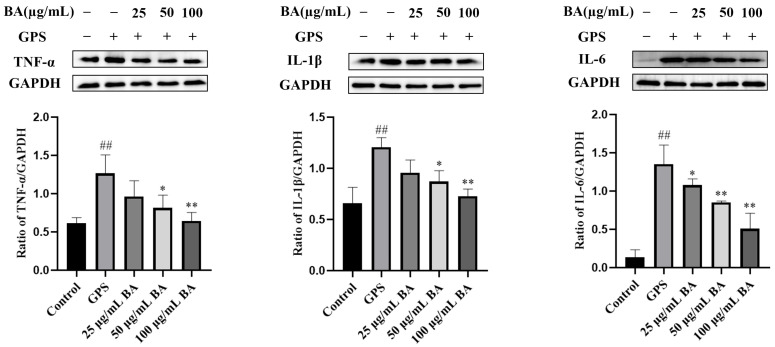
Baicalin inhibited the expression of inflammatory proteins in porcine peritoneal mesothelial cells infected with *Glaesserella parasuis*. Baicalin reduced the upregulation of TNF-α, IL-1β, and IL-6. *GPS* stands for *Glaesserella parasuis*; BA stands for baicalin; TNF-α stands for tumor necrosis factor alpha; IL-1β stands for interleukin-1 beta; IL-6 stands for interleukin-6; GAPDH stands for glyceraldehyde-3-phosphate dehydrogenase. Significant differences are denoted as follows: ## represents *p* < 0.01 for the comparison between the control group and the *GPS* infection group; * and ** represent *p* < 0.05 and *p* < 0.01, respectively, for the comparisons between the *GPS* infection group and the baicalin groups.

The Western blot assay was used to explore the effect of baicalin on the expression of inflammatory proteins in PPMCs infected with *GPS*. Compared to the control group, the protein expressions of TNF-α, IL-1β, and IL-6 in the *GPS* infection group were significantly higher (*p* < 0.01). Compared to the *GPS* infection group, the expression of IL-6 in the 25 μg/mL baicalin group was significantly lower (*p* < 0.05), while the expressions of IL-1β and TNF-α were insignificantly lower (*p* > 0.05). The expression of IL-6 in the 50 μg/mL and 100 μg/mL baicalin groups was significantly lower (*p* < 0.01), as were the expressions of IL-1β and TNF-α (*p* < 0.05). The expressions of IL-1β and TNF-α in the 100 μg/mL baicalin group were significantly lower (*p* < 0.01) (Figure 2).

### 3.3. Baicalin Inhibition of the Expression of ADAM17/EGFR Axis-Related Proteins in PPMCs Infected with GPS

**Figure 3:**
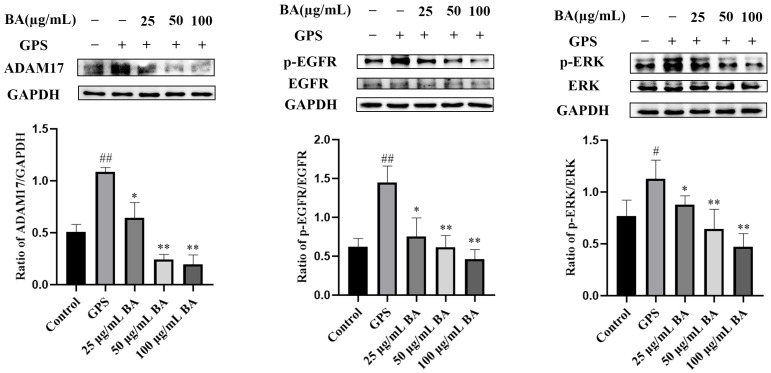
Baicalin inhibited the expression of ADAM17/EGFR axis-related proteins in porcine peritoneal mesothelial cells infected with *Glaesserella parasuis*. Baicalin reduced the upregulation of ADAM17, p-EGFR/EGFR, and p-ERK/ERK. *GPS* stands for *Glaesserella parasuis*; BA stands for baicalin; ADAM17 stands for disintegrin and metalloproteinase 17; EGFR stands for epidermal growth factor receptor; p-EGFR stands for phosphorylated EGFR; ERK stands for extracellular signal-regulated kinase; p-ERK stands for phosphorylated ERK; GAPDH stands for glyceraldehyde-3-phosphate dehydrogenase. Significant differences are denoted as follows: # and ## represent *p* < 0.05 and *p* < 0.01, respectively, for the comparisons between the control group and the *GPS* infection group; * and ** represent *p* < 0.05 and *p* < 0.01, respectively, for the comparisons between the *GPS* infection group and the baicalin groups.

The effects of baicalin on the expression of ADAM17/EGFR axis-related proteins in PPMCs infected with *GPS* are shown in Figure 3. The expression of ADAM17 proteins in the *GPS*-infected group was significantly higher than in the control group (*p* < 0.01). Compared to the *GPS*-infected group, the expressions of ADAM17 in the 25 μg/mL, 50 μg/mL, and 100 μg/mL baicalin groups were significantly decreased (*p* < 0.05). Moreover, compared to the control group, significant increases in the expressions of p-EGFR/EGFR (*p* < 0.01) and p-ERK/ERK (*p* < 0.05) were observed in the *GPS*-infected group. Compared to the *GPS*-infected group, the expressions of p-EGFR/EGFR and p-ERK/ERK in the 25 μg/mL baicalin group were significantly decreased (*p* < 0.05); the protein expressions of p-EGFR/EGFR and p-ERK/ERK in the 50 μg/mL and 100 μg/mL baicalin groups were also significantly decreased (*p* < 0.01).

### 3.4. Baicalin Inhibition of Inflammatory Protein Expression via the ADAM17/EGFR Axis in PPMCs Infected with GPS

**Figure 4:**
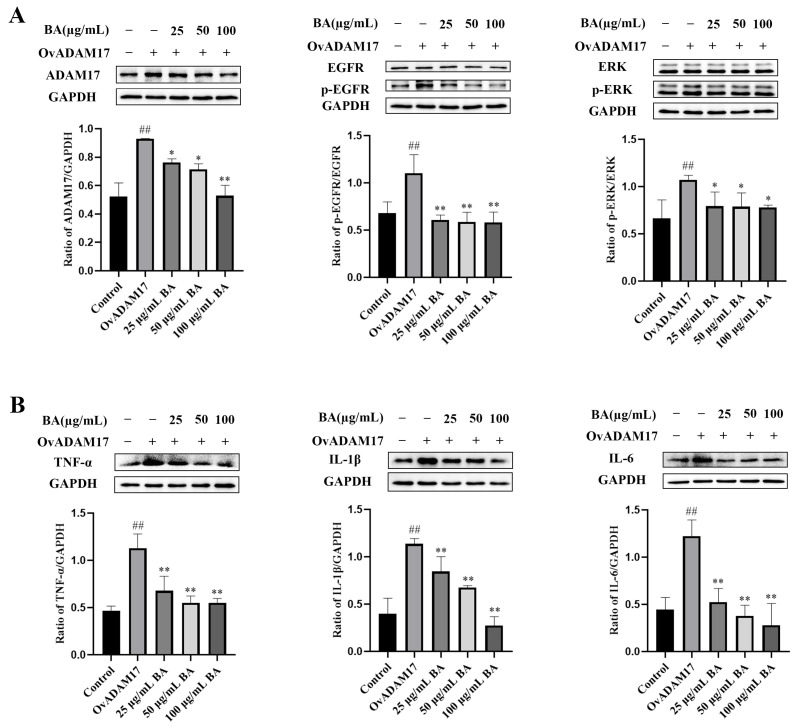
Baicalin inhibited the expression of ADAM17/EGFR axis-related proteins and inflammatory proteins induced by ADAM17 overexpression in porcine peritoneal mesothelial cells. (**A**) Baicalin reduced the upregulation of ADAM17, p-EGFR/EGFR, and p-ERK/ERK. (**B**) Baicalin reduced the upregulation of TNF-α, IL-1β, and IL-6. OvADAM17 stands for overexpression of ADAM17; BA stands for baicalin; ADAM17 stands for disintegrin and metalloproteinase 17; EGFR stands for epidermal growth factor receptor; p-EGFR stands for phosphorylated EGFR; ERK stands for extracellular signal-regulated kinase; p-ERK stands for phosphorylated ERK; TNF-α stands for tumor necrosis factor alpha; IL-1β stands for interleukin-1 beta; IL-6 stands for interleukin-6; GAPDH stands for glyceraldehyde-3-phosphate dehydrogenase. Significant differences are denoted as follows: ## represents *p* < 0.01 for the comparison between the control group and the OvADAM17 group; * and ** represent *p* < 0.05 and *p* < 0.01, respectively, for the comparisons between the OvADAM17 group and the baicalin groups.

**Figure 5:**
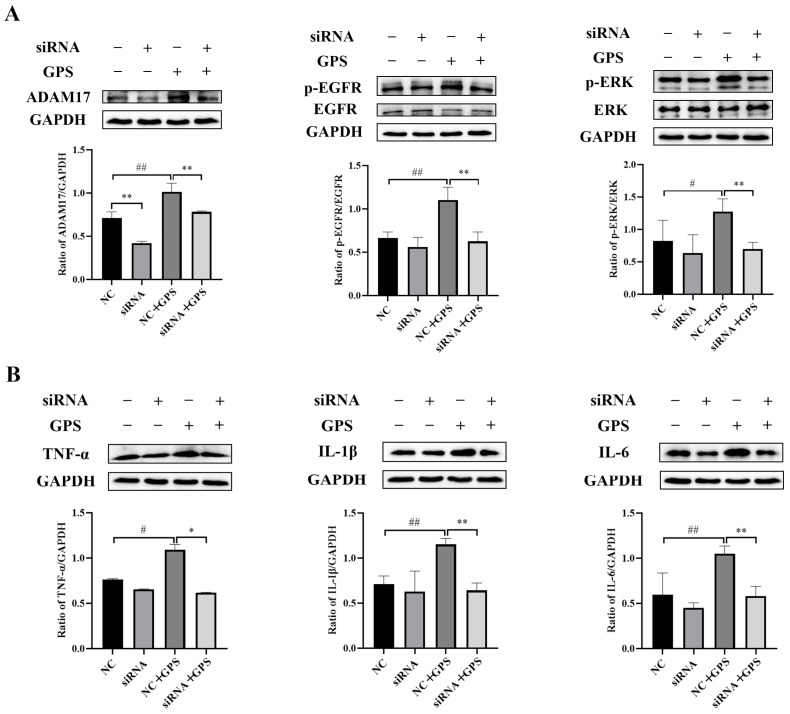
ADAM17 small interfering RNA (siRNA) inhibited the expression of ADAM17/EGFR axis-related proteins and inflammatory proteins in porcine peritoneal mesothelial cells infected by *Glaesserella parasuis*. (**A**) ADAM17 siRNA reduced the upregulation of ADAM17, p-EGFR/EGFR, and p-ERK/ERK. (**B**) ADAM17 siRNA reduced the upregulation of TNF-α, IL-1β, and IL-6. NC stands for negative control siRNA; siRNA stands for ADAM17 siRNA; *GPS* stands for *Glaesserella parasuis*; ADAM17 stands for disintegrin and metalloproteinase 17; EGFR stands for epidermal growth factor receptor; p-EGFR stands for phosphorylated EGFR; ERK stands for extracellular signal-regulated kinase; p-ERK stands for phosphorylated ERK; TNF-α stands for tumor necrosis factor alpha; IL-1β stands for interleukin-1 beta; IL-6 stands for interleukin-6; GAPDH stands for glyceraldehyde-3-phosphate dehydrogenase. Significant differences are denoted as follows: # and ## represent *p* < 0.05 and *p* < 0.01, respectively, for the comparisons between the negative control siRNA group and the negative control siRNA plus *GPS* group; * and ** represent *p* < 0.05 and *p* < 0.01, respectively, for the comparisons between the corresponding negative control siRNA and ADAM17 siRNA groups, both in the presence and absence of *GPS* infection.

The inhibition of inflammatory protein expression through the ADAM17/EGFR axis by overexpression of ADAM17 (OvADAM17) and ADAM17 siRNA in PPMCs infected with GPS is shown in Figure 4 and Figure 5. Compared to the control group, the protein expressions of ADAM17, p-EGFR/EGFR, p-ERK/ERK, TNF-α, IL-1β, and IL-6 in the OvADAM17 group were significantly increased (*p* < 0.01) (Figure 4). However, with the administration of 25–100 μg/mL of baicalin, the expressions of these proteins were significantly inhibited to varying degrees (*p* < 0.05). Compared to the negative control siRNA group, the expressions of ADAM17, p-EGFR/EGFR, p-ERK/ERK, TNF-α, IL-1β, and IL-6 in the negative control siRNA plus *GPS* group were significantly increased (*p* < 0.05) (Figure 5). However, with the intervention of ADAM17 siRNA, the expressions of these proteins were significantly inhibited to varying degrees (*p* < 0.05).

### 3.5. Baicalin Inhibition of Inflammatory Protein Expression via the ADAM17/EGFR Axis in the Peritoneum of Piglets Infected with GPS

**Figure 6:**
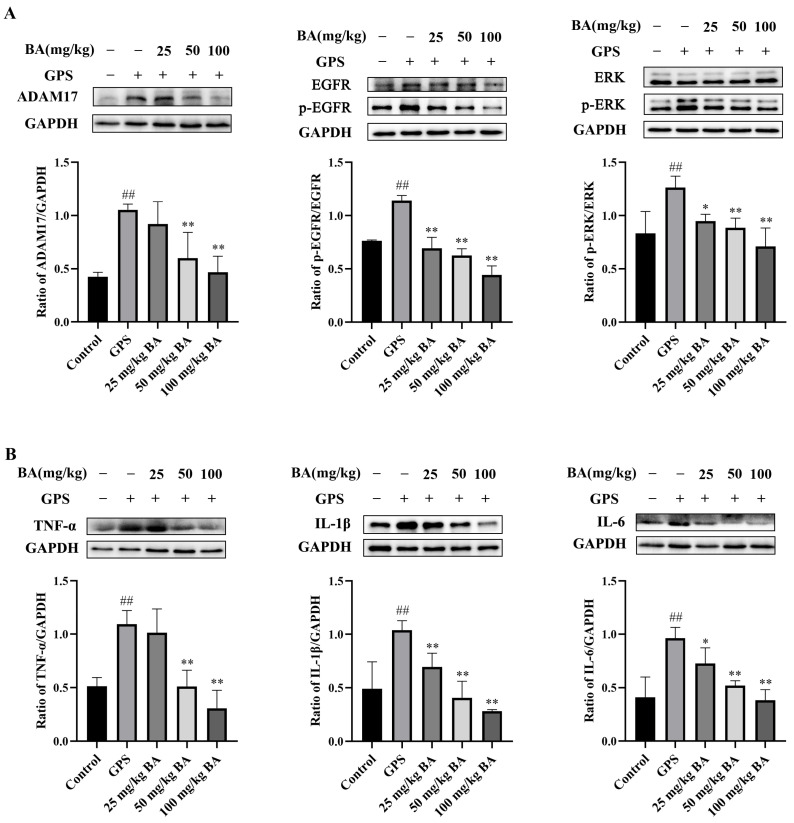
Baicalin alleviated inflammatory protein expression via the ADAM17/EGFR axis in the peritoneum of piglets infected with *Glaesserella parasuis*. (**A**) Baicalin reduced the upregulation of ADAM17, p-EGFR/EGFR, and p-ERK/ERK. (**B**) Baicalin reduced the upregulation of TNF-α, IL-1β, and IL-6. *GPS* stands for *Glaesserella parasuis*; BA stands for baicalin; ADAM17 stands for disintegrin and metalloproteinase 17; EGFR stands for epidermal growth factor receptor; p-EGFR stands for phosphorylated EGFR; ERK stands for extracellular signal-regulated kinase; p-ERK stands for phosphorylated ERK; TNF-α stands for tumor necrosis factor alpha; IL-1β stands for interleukin-1 beta; IL-6 stands for interleukin-6; GAPDH stands for glyceraldehyde-3-phosphate dehydrogenase. Significant differences are denoted as follows: ## represents *p* < 0.01 for the comparison between the control group and the *GPS* challenge group; * and ** represent *p* < 0.05 and *p* < 0.01, respectively, for the comparisons between the *GPS* challenge group and the baicalin treatment groups.

Compared to the control group, the protein expressions of ADAM17, p-EGFR/EGFR, p-ERK/ERK, TNF-α, IL-1β, and IL-6 in the peritoneum of piglets infected with *GPS* were significantly higher (*p* < 0.01) (Figure 6). Compared to the *GPS* challenge group, the 50 μg/mL and 100 μg/mL baicalin treatment groups showed significantly (*p* < 0.01) reduced expression of ADAM17 and TNF-α. Moreover, the three baicalin treatment groups significantly reduced the expression of p-EGFR/EGFR, p-ERK/ERK, IL-1β, and IL-6 (*p* < 0.05) compared to the *GPS* challenge group.

## 4. Discussion

*GPS* is an opportunistic bacterium that can be localized in the upper respiratory tract of healthy piglets and cause Glässer’s disease, with high morbidity and mortality under stress conditions [29,30]. Under stress conditions, *GPS*-infected piglets suffer severe systemic inflammation characterized by fibrinous polyserositis, meningitis, pneumonia, and arthritis [31,32,33]. However, the mechanism of peritonitis from the perspective of inflammation remains unclear. In this study, we investigated the regulatory effect of baicalin on inflammatory injury of peritonitis caused by *GPS* infection in vivo and in vitro. Our results provide an effective option to prevent and control peritonitis caused by *GPS* infection.

*GPS* in piglets can induce peritoneal injury and promote peritonitis. The infection of piglets with *GPS* induced the characteristic polyserositis with mixed inflammatory exudate in peritoneal cavities [34]. We also observed this phenomenon in an earlier study [12] and illustrated that *GPS* could disrupt the expression of tight junctions’ genes in the peritoneum of *GPS*-infected piglets and induce cell apoptosis via the PKC-MAPK pathway in PPMCs. Baicalin can alleviate the apoptosis and tight junction injury of peritonitis induced by *GPS* infection [3,12]. However, it has not been clarified whether or not baicalin can alleviate peritonitis induced by *GPS* in piglets by alleviating inflammatory injury. Our results revealed that baicalin can decrease the protein expressions of TNF-α, IL-1β, and IL-6 in *GPS*-infected PPMCs and piglets, which suggests that the regulation of inflammatory injury is helpful in alleviating peritonitis caused by *GPS*.

The ADAM17/EGFR axis plays an important role in regulating inflammatory injury in diseases. In the context of inflammation, ADAM17 acts as an integral signal regulator by mediating the release of membrane-anchored cytokines, cell adhesion molecules, membrane receptors, and enzymes [35,36,37]. ADAM17 also acts as a cleaving enzyme to activate the high-affinity EGFR ligand-transforming growth factor alpha, which mediates EGFR phosphorylation and further activates the EGFR–ERK signaling cascade [38,39]. EGFR–ERK signaling contributes to the expression of various pro-inflammatory proteins [40,41,42]. The loss of ADAM17 greatly reduced the release of TNF-α and IL-6 and pulmonary leukocyte recruitment in an endotoxin-induced acute lung injury model [43], and it also inhibited the expression of p-EGFR in colonic inflammation [44] and the phosphorylation of EGFR and proinflammatory factor IL-8 expression in lipopolysaccharide-treated A549 lung epithelial cells [45]. ADAM17 can induce the inflammatory injury of vascular smooth muscle cells through the EGFR-ERK pathway, and ADAM17 siRNA can significantly inhibit the expression of the inflammatory factors IL-1β, IL-6, and TNF-α, as well as p-EGFR and p-ERK [46]. In our study, baicalin alleviated the ADAM17/EGFR axis-induced inflammatory injury of peritonitis in PPMCs and piglets infected by *GPS*, which means that targeting ADAM17 may be the key to alleviating the inflammatory injury of piglet peritonitis.

This study has several limitations. First, the findings are specific to the *GPS* serovar tested; their generalizability to other serovars, which is crucial given the known vaccine cross-protection issues, remains unverified. Second, the short 7-day in vivo observation period may not capture long-term therapeutic effects or the development of potential chronic inflammatory responses. Third, although a dose–response relationship for baicalin was observed, the specific dosage range tested may not represent the optimal therapeutic window, warranting further dose range and route studies. Fourth, while our data strongly suggest an association between baicalin and the downregulation of the ADAM17/EGFR/ERK pathway, the experimental design did not include definitive rescue experiments (e.g., utilizing EGFR agonists) to unequivocally establish the causality for this specific pathway in mediating baicalin’s effects observed here. Furthermore, although we focused on ADAM17, we acknowledge the possibility that other metalloproteinases might contribute to the observed effects or exhibit functional redundancy; our study did not specifically investigate or rule out this potential contribution. Fifth, although our previous work demonstrated baicalin’s dose-dependent improvement of key clinical outcomes (survival, weight change, temperature) [28] and this study confirms its capacity to alleviate peritoneal inflammatory injury across doses, its practical application as an anti-inflammatory agent—including optimal formulation, administration route across species, clinical efficacy confirmation, and potential as an antibiotic alternative—remains unexplored and requires future research.

## 5. Conclusions

Our study shows that baicalin has a potential therapeutic effect on *GPS*-induced piglet peritonitis. Baicalin is associated with the suppression of inflammatory factor expression, likely via modulation of the ADAM17/EGFR axis, and the improvement of the peritonitis injury caused by *GPS* infection in piglets. Elucidating the regulatory role of baicalin in peritonitis provides a theoretical basis for preventing and controlling *GPS* infection and developing novel non-antibiotic strategies, including phytochemical therapeutics and feed additives.

## Data Availability

The data presented in this study are available in the article.

## Abbreviations

The following abbreviations are used in this manuscript:

ADAM17	Disintegrin and metalloproteinase 17
BSA	Bovine serum albumin
b.w.	Body weight
CCK8	Cell counting kit-8
CFUs	Colony-forming units
EGFR	Epidermal growth factor receptor
ERK	Extracellular signal-regulated kinase
GAPDH	Glyceraldehyde-3-phosphate dehydrogenase
GPS	Glaesserella parasuis
HMGB1	High mobility group box 1
IL-1β	Interleukin-1 beta
IL-6	Interleukin-6
NC	Negative control siRNA
NF-κB	Nuclear factor kappa B
NLRP3	NOD-like receptor pyrin 3 inflammasome
OvADAM17	Overexpression of ADAM17
PKC-MAPK	Protein kinase C-mitogen-activated protein kinases
PPMCs	Porcine peritoneal mesothelial cells
siRNA	Small interfering RNA
TBST	Tris-buffered saline containing 0.1% Tween-20
TNF-α	Tumor necrosis factor alpha
TSB	Tryptic soy broth
